# Dapagliflozin rescues endoplasmic reticulum stress-mediated cell death

**DOI:** 10.1038/s41598-019-46402-6

**Published:** 2019-07-08

**Authors:** Ryo Shibusawa, Eijiro Yamada, Shuichi Okada, Yasuyo Nakajima, Claire C. Bastie, Akito Maeshima, Kyoichi Kaira, Masanobu Yamada

**Affiliations:** 1 Department of Medicine and Molecular Science, Gunma University Graduate School of Medicine, Maebashi, 371-8511 Japan; 2 Division of Biomedical Sciences, Warwick Medical School, Coventry, West Midlands United Kingdom; 3 Division of Nephrology, Department of Internal Medicine, Jichi Medical University, Shimotsuke, Tochigii Japan; 4 Department of Oncology Clinical Development, Gunma University Graduate School of Medicine, Maebashi, 371-8511 Japan

**Keywords:** Cell death, Diabetes complications

## Abstract

The new type 2 diabetes drug, dapagliflozin, reduces blood glucose levels and body weight by inhibiting sodium glucose transporter 2 (SGLT2) in proximal tubular cells. SGLT2 inhibitors might modulate glucose influx into renal tubular cells, thereby regulating the metabolic conditions that cause endoplasmic reticulum (ER) stress in the cells. In this study, we examined the effect of dapagliflozin on ER stress in the HK-2 proximal tubular cell line and in the kidney of db/db mice to characterise its function in diabetic nephropathy (DN). We found that dapagliflozin regulated ER stress-mediated apoptosis *in vitro* and *in vivo*. Only the elf2α-ATF4-CHOP pathway was regulated under these conditions. Notably, the drug rescued C2 ceramide-induced ER stress-mediated apoptosis and ER stress-mediated apoptosis, which might occur in DN, in db/db mice. Our study shows a novel role for dapagliflozin as an inhibitor of ER stress and suggests that dapagliflozin might be useful for the prevention of DN.

## Introduction

The number of patients with diabetes has increased globally, partly due to changes in dietary habits and a lack of exercise^1–3^. Diabetes leads to an array of additional complications, and the disease and its prevention have become a worldwide priority^4^. One of the major complications of diabetes is diabetic nephropathy (DN), which is the leading cause of end-stage renal disease^5,6^. Several studies have shown that the diabetic kidney is exposed to various environmental stressors that may cause endoplasmic reticulum (ER) stress^5,6^.

The ER is the major site for protein folding^7,8^. When protein loading exceeds its capacity, unfolded protein response (UPR) is activated to suppress protein synthesis and increase both the ER folding capacity and misfolded protein degradation, resulting in the restoration of cellular homeostasis^7^. If the stress response is prolonged or beyond ER folding capacity, it may lead to cell death^7^. UPR is mediated by three canonical pathways: RNA-dependent protein kinase-like ER kinase (PERK), activating transcription factor 6 (ATF6), and inositol-requiring enzyme 1 (IRE1) pathways^7^. PERK is present in the ER and its phosphorylation at Ser51 of eukaryotic translation initiation factor 2α (eIF2α) inhibits protein translation^7,9^. Notably, ATF4 activated by phosphorylation of eIF2α initiates the transcription of CCAAT/enhancer-binding protein-homologous protein (Chop/Gadd153), which is critical in ER stress-induced apoptosis^7,9,10^. Although ATF6 also exists in the ER, it is transported to the Golgi apparatus under stress conditions and cleaved by site-1 and site-2 proteases^7,11^. Cleaved ATF6 enters the nucleus and transcribes adaptive chaperons such as Bip/GRP78^7,11^. IRE1 exerts endoribonuclease and kinase activities^7,12^. The endoribonuclease activity of IRE1 cleaves the X-box binding protein 1 (XBP1) to generate spliced XBP1 (XBP1s), a transcription factor involved in the survival response^7,12^. It has been shown that the IRE1α-XBP1 pathway plays a role in insulin resistance by activating JNK and suppressing insulin signalling; however, the relationship between ER stress and diabetic complications is still largely unknown^13–15^.

Ceramides are important molecules involved in sphingolipid metabolism and play critical roles in the regulation of various cellular functions, including cell proliferation, differentiation, migration, and apoptosis^16,17^. The short side chain C2 ceramide easily invades cells, exerts physiological activity, and enhances caspase-dependent apoptosis^17^. It has been shown that plasma C2 ceramide levels also increase in diabetes^18^. They play an important pathophysiological role not only in the development of diabetes itself but also in diabetic complications^19–22^. C2 ceramide induces apoptosis by multiple mechanisms, namely activation of the extrinsic apoptotic pathway, increasing cytochrome c release, generation of free radicals, and induction of ER stress^19,20^.

Glomerular damage is considered the main characteristic in the kidneys of patients with DN. However, it was recently suggested that alterations in the renal tubule might also contribute to the pathogenesis of DN^23^. Notably, the novel type 2 diabetes drug dapagliflozin reduces blood glucose and body weight by inhibiting sodium glucose transporter 2 (SGLT2) in the proximal tubular cells^24^. We hypothesised that modulation of SGLT2 activity might affect glucose influx into renal tubular cells. Inhibiting SGLT2 with dapagliflozin may regulate the metabolic conditions that cause ER stress in these cells^25^. Therefore, in this study, we examined the effect of the SGLT2 inhibitor dapagliflozin on UPR in both the *in vitro* proximal tubular cell line HK-2 and *in vivo*.

## Results

### Dapagliflozin mediates glucose influx and ER stress in HK2 cells

**Figure 1:**
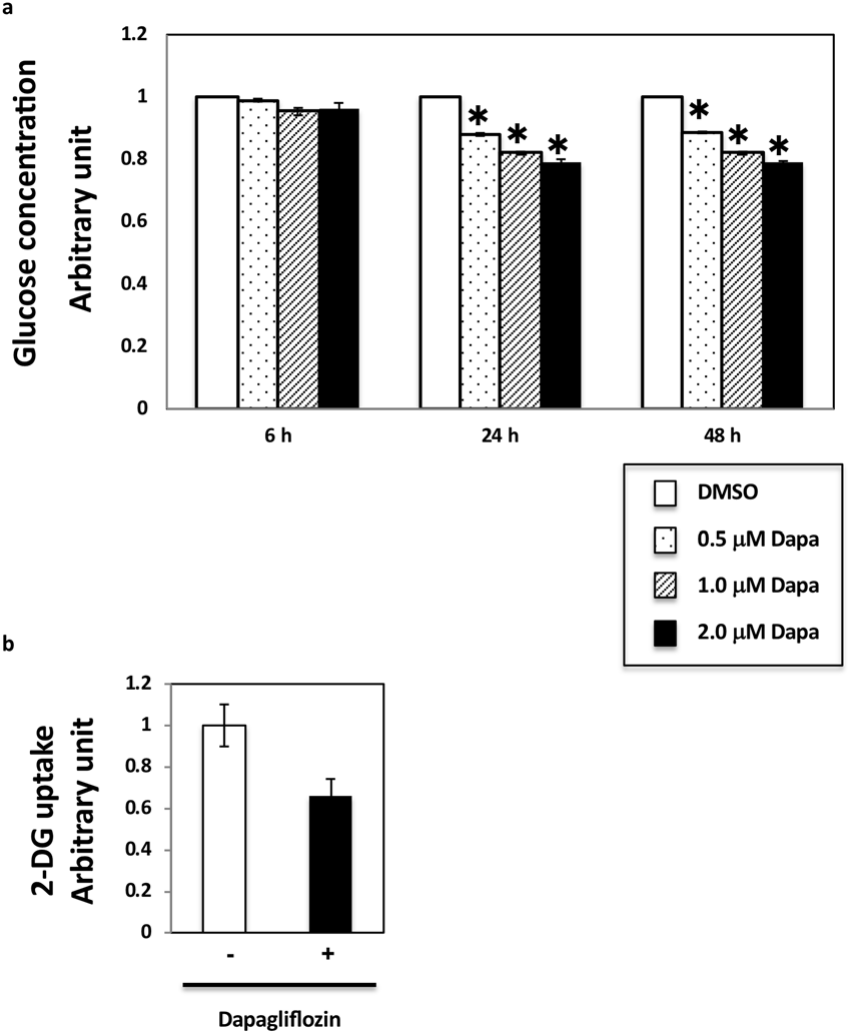
Dapagliflozin regulates glucose influx into HK-2 renal proximal tubular epithelial cells. (**a**) HK2 cells were incubated with the indicated dapagliflozin concentration for the indicated times and were collected for glucose analysis. Glucose concentration in HK2 cells was assessed using the PicoProbe Glucose Fluorometric Assay Kit (BioVision). P < 0.05 (n = 3–5). (**b**) Glucose influx in HK2 cells incubated with 2 µM dapagliflozin for 36 h was measured using the 2-Deoxyglucose Uptake Measurement Kit (COSMO BIO Co. Ltd., Tokyo Japan) according to the manufacturer’s instructions.

**Figure 2:**
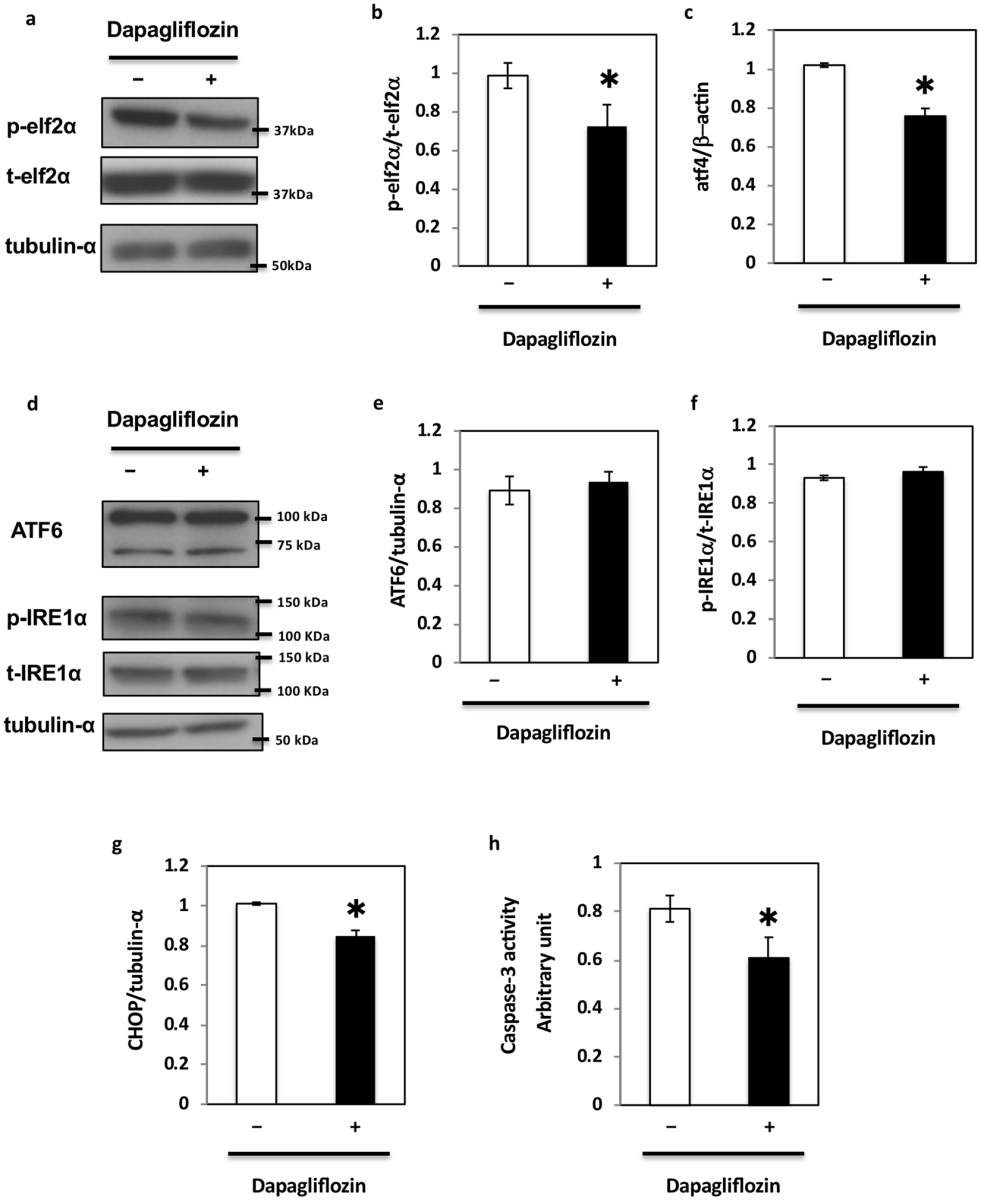
Dapagliflozin regulates unfolded protein response in HK-2 cells. (**a**) HK2 cells were incubated with 2 μM dapagliflozin for 36 h and collected for protein expression analysis. These immunoblots are representative of n = 5 experiments performed independently. (**b**) Signal quantification of the expression levels for S^51^ phosphorylation of elf2α normalised with total elf2α levels. (**c**) HK2 cells were incubated with 2 μM dapagliflozin for 36 h, and the ATF4 mRNA expression levels were determined by qRT-PCR (n = 5 independent experiments). (**d**) HK2 cells were incubated with 2 μM dapagliflozin for 36 h, collected for protein analysis, and immunoblotted for the indicated proteins. These are representative immunoblots independently performed five times. (**e**,**f**) Signal quantification of the expression levels of ATF6 normalised with tubulin-α (**e**) and the expression levels for S^724^ phosphorylation of IRE1α normalised with total IRE1α levels (**f**). (**g**) HK2 cells were incubated with 2 μM dapagliflozin for 36 h, and the expression level of CHOP mRNA was determined by qRT-PCR (n = 5 independent experiments). (**h**) HK2 cells were incubated with 2 μM dapagliflozin for 36 h and caspase-3 activity levels were measured using a caspase-3 fluorescence assay kit (MBL) (n = 5 independent experiments).

Dapagliflozin inhibits SGLT2-mediated renal glucose reabsorption in renal proximal tubular epithelial cells, thereby lowering the plasma blood glucose levels in patients with diabetes^10,12,13^. To identify additional functions of SGLT2, we used HK2 cells, an *in vitro* cell model of renal proximal tubular epithelial cells. First, we performed a series of dose-response and time course experiments to determine the effects of dapagliflozin on glucose levels in HK2 cells. Since a maximum plasma concentration (Cmax) of dapagliflozin of approximately 0.5 μM and 0.2–20 μM was used in previous studies, we initially choose 0.5–2.0 μM dapagliflozin. We found that intracellular glucose concentrations in cells treated with 2 μM dapagliflozin for 24–48 h decreased by 20%, compared to those in control (non-treated) cells (Fig. 1a). Since the effect of dapagliflozin was maximum at this condition, we decided to use it for all *in vitro* experiments. Notably, glucose influx measured with tracers decreased by around 40% under this condition (Fig. 1b). As glucose influx could potentially mediate ER stress in cells and tissues, we next examined the markers of ER stress in dapagliflozin-treated HK-2 cells. We first evaluated the basal level of ER stress. Since the basal level of ER stress might depend on cell confluency, we plated different cell numbers on 24 wells and found that the phosphorylation of elf2α, a marker for ER stress, was the maximum 48 h after we plated 1 × 10^6^ cells in 24 wells (Supplementary Fig. 1). Under these conditions, dapagliflozin decreased the phosphorylation of elf2α (Fig. 2a,b). To confirm this, we examined the downstream targets of elf2α. As shown in Fig. 2c, the decrease in ATF4 expression levels in dapagliflozin-treated HK2 cells was parallel to the decrease in eIf2α phosphorylation levels. In contrast, dapagliflozin had no effect on total and cleaved forms of ATF6 (Fig. 2d,e) or phosphorylation of IRE1α (Fig. 2d,f), indicating that it mediated ER stress only through the elf2α pathway.

CHOP has recently been reported as a downstream target of elf2α and has been described as a marker of ER-mediated apoptosis^7,9,10^. As shown in Fig. 2g, CHOP expression levels also decreased in dapagliflozin-treated HK2 cells. Because CHOP expression levels decreased, we next examined caspase-3 activity to determine the apoptotic status of these cells. As shown in Fig. 2h, we observed a 20% reduction in caspase-3 activity in dapagliflozin-treated cells. This paralleled the reduction in glucose influx, phosphorylation of elF2α, and expression of CHOP, suggesting that dapagliflozin might regulate apoptosis through the elf2α-CHOP axis in HK2 cells.

### The eIF2α pathway is critical for the effects of dapagliflozin on ER stress in HK2 cells

**Figure 3:**
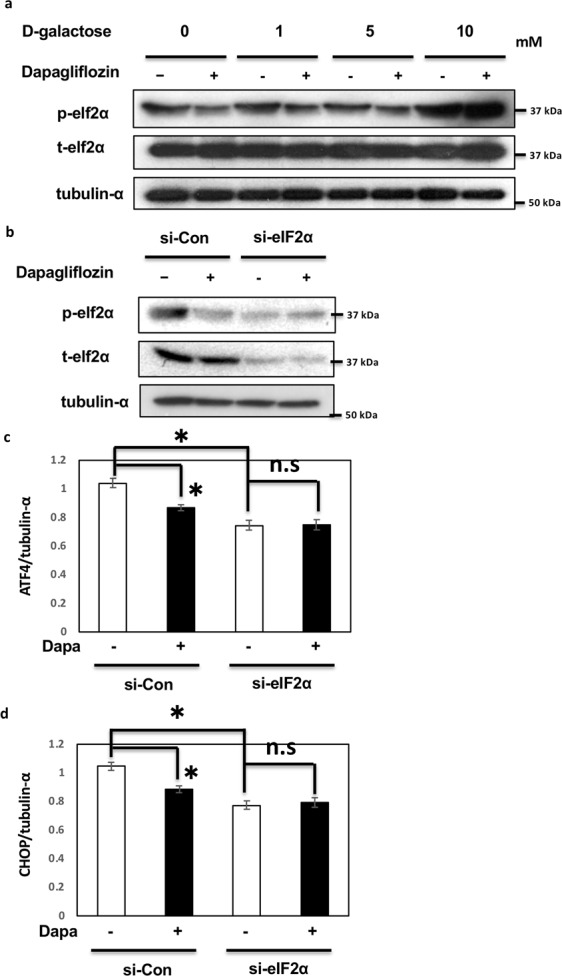
Dapagliflozin regulates unfolded protein response through the eIF2α pathway in HK-2 cells. (**a**) HK2 cells were treated with 2 μM dapagliflozin for 36 h with varying concentrations of D-galactose. Immunoblotting was performed with the indicated antibodies. (**b**) HK2 cells were transfected by siRNA for elf2α and incubated with 2 μM dapagliflozin for 36 h. Immunoblotting was performed with the indicated antibodies. (**c**,**d**) HK2 cells were transfected by siRNA for elf2α and incubated with 2 μM dapagliflozin for 36 h. The mRNA expression levels of ATF4 and CHOP were determined by qRT-PCR (n = 5 independent experiments).

To examine whether the effects of dapagliflozin were the result of reduced glucose uptake or a direct effect of dapagliflozin, the medium of HK-2 cells was supplemented with different concentrations (0–10 mM) of D-galactose before the analyses of elf2α and CHOP expression. As shown in Fig. 3a, dapagliflozin reduced the phosphorylation of elf2α, except in the presence of 10 mM D-galactose, indicating that the effect of dapagliflozin was due to reduced glucose uptake. To investigate whether the elf2α pathway is critical for the effect of dapagliflozin on ER stress, siRNA-mediated knockdown of elf2α was performed, and ATF4 and CHOP expression was determined. As shown in Fig. 3c,d, siRNA-mediated knockdown of elf2α reduced the basal levels of ATF4 and CHOP. Notably, the effects of dapagliflozin on ATF4 and CHOP were completely abolished under these conditions, indicating that the elf2α pathway plays a crucial role in mediating the effects of dapagliflozin (Fig. 3c,d).

### Dapagliflozin rescued C2 ceramide-induced ER stress through the elf2α-CHOP pathway

**Figure 4:**
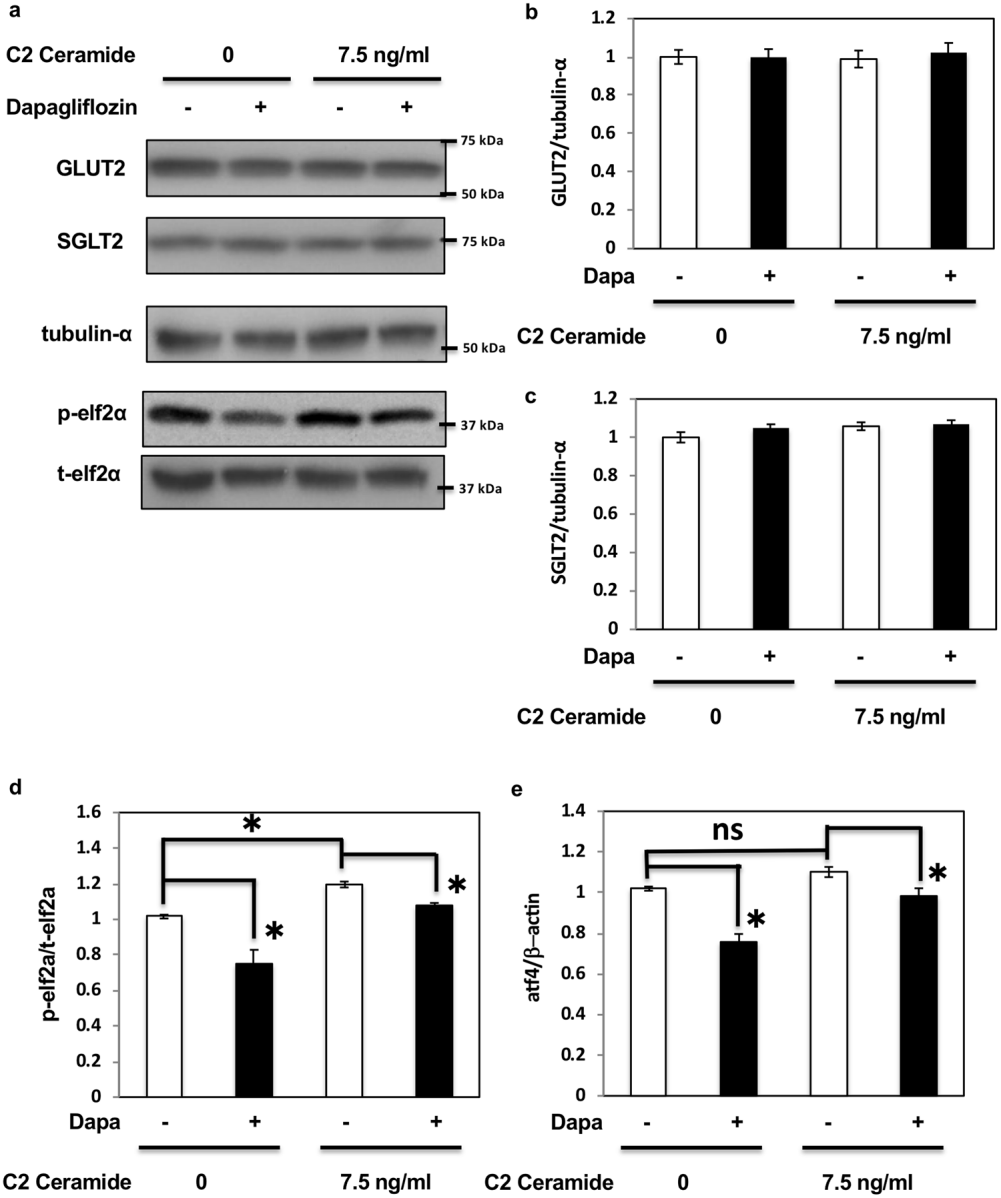
Dapagliflozin rescued C2 ceramide-induced ER stress in HK2 cells. (**a**) HK2 cells were treated with 2 μM dapagliflozin for 36 h with or without 7.5 ng/mL C2 ceramide, and immunoblotting was performed with the indicated antibodies (**b**,**c**). Signal quantification of the expression levels of GLUT2 (**b**) and SGLT2 (**c**) normalised with tubulin-α levels. (**d**) Signal quantification of the expression levels of S^51^ phosphorylation of elf2α normalised with total elf2α levels. (**e**) HK2 cells were incubated with 2 μM dapagliflozin for 36 h, and the mRNA expression levels of ATF4 were determined by qRT-PCR (n = 5 independent experiments).

**Figure 5:**
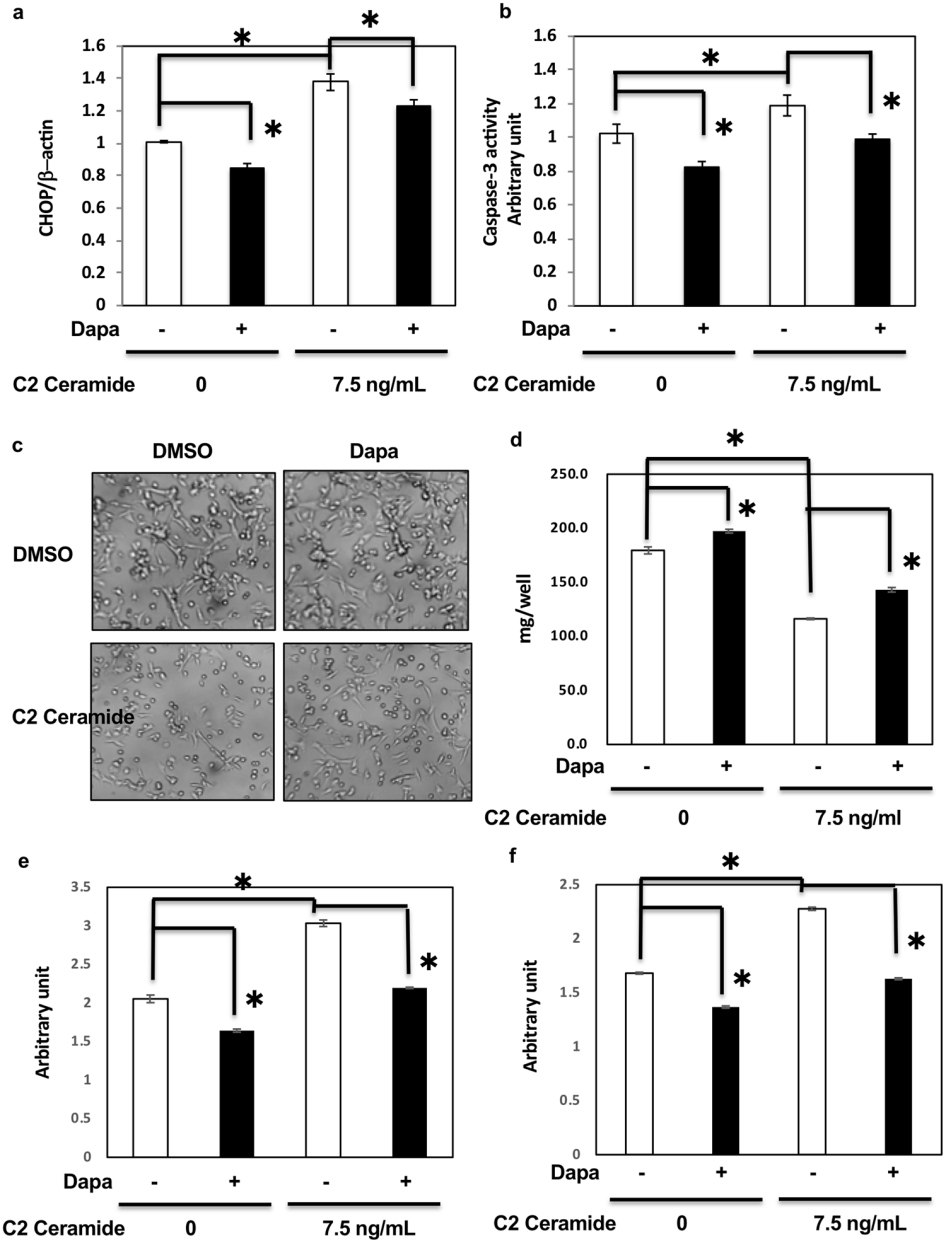
Dapagliflozin rescued C2 ceramide-induced cell death in HK2 cells. (**a**) HK2 cells were incubated with 2 μM dapagliflozin for 36 h with or without 7.5 ng/ml C2 ceramide, and the expression levels of CHOP mRNA were determined by qRT-PCR (n = 5 independent experiments). (**b**) HK2 cells were treated with 2 μM dapagliflozin for 36 h with or without 7.5 ng/ml C2 ceramide, and caspase-3 activity levels were determined (n = 5 independent experiments). (**c**,**d**) HK2 cells were treated with 2 μM dapagliflozin for 48 h with or without 7.5 ng/ml C2 ceramide, and the cells were subjected to microscopic observation (**c**) and protein assay (**d**). These are representative images from experiments independently performed five times. (**e**), (**f**) HK2 cells were treated with 2 μM dapagliflozin for 48 h with or without 7.5 ng/ml C2 ceramide, and apoptosis and necrosis assay was performed using a Cell Death Detection ELISAplus (Roche, Indianapolis, IN).

Our data suggested that dapagliflozin mediated apoptosis through ER stress; however, whether dapagliflozin could be a key factor in the prevention of diabetic kidney disease remained to be determined. Therefore, to confirm the effect of dapagliflozin on ER stress and to examine its effect on the kidney in metabolic diseases, we used C2 ceramide, which not only induces ER stress but also acts as a pathogen in metabolic diseases^19–22^. As shown in Fig. 4, dapagliflozin had no effect on GLUT2 and SGLT2 expression (Fig. 4a–c) but decreased elf2α phosphorylation (Fig. 4a,d) and ATF4 expression (Fig. 4e). C2 ceramide enhanced elf2α phosphorylation (Fig. 4a,d) and ATF4 expression (Fig. 4e). Dapagliflozin partially reduced these effects, suggesting that it might alleviate C2 ceramide-induced ER stress. Notably, C2 ceramide-induced CHOP expression and caspase-3 activity were also inhibited by dapagliflozin (Fig. 5a,b). C2 ceramide reduced cell number and protein amounts in HK-2 cells, indicating cell death. However, dapagliflozin rescued the effect of C2 ceramide (Fig. 5c,d) by regulating apoptosis and necrosis (Fig. 5e,f). These data indicate that dapagliflozin might attenuate the metabolic state that induces renal cell death and exhibit beneficial effects in preventing DN, independently of blood glucose reduction.

### Dapagliflozin ameliorates ER stress in the kidney of db/db mice *in vivo*

**Figure 6:**
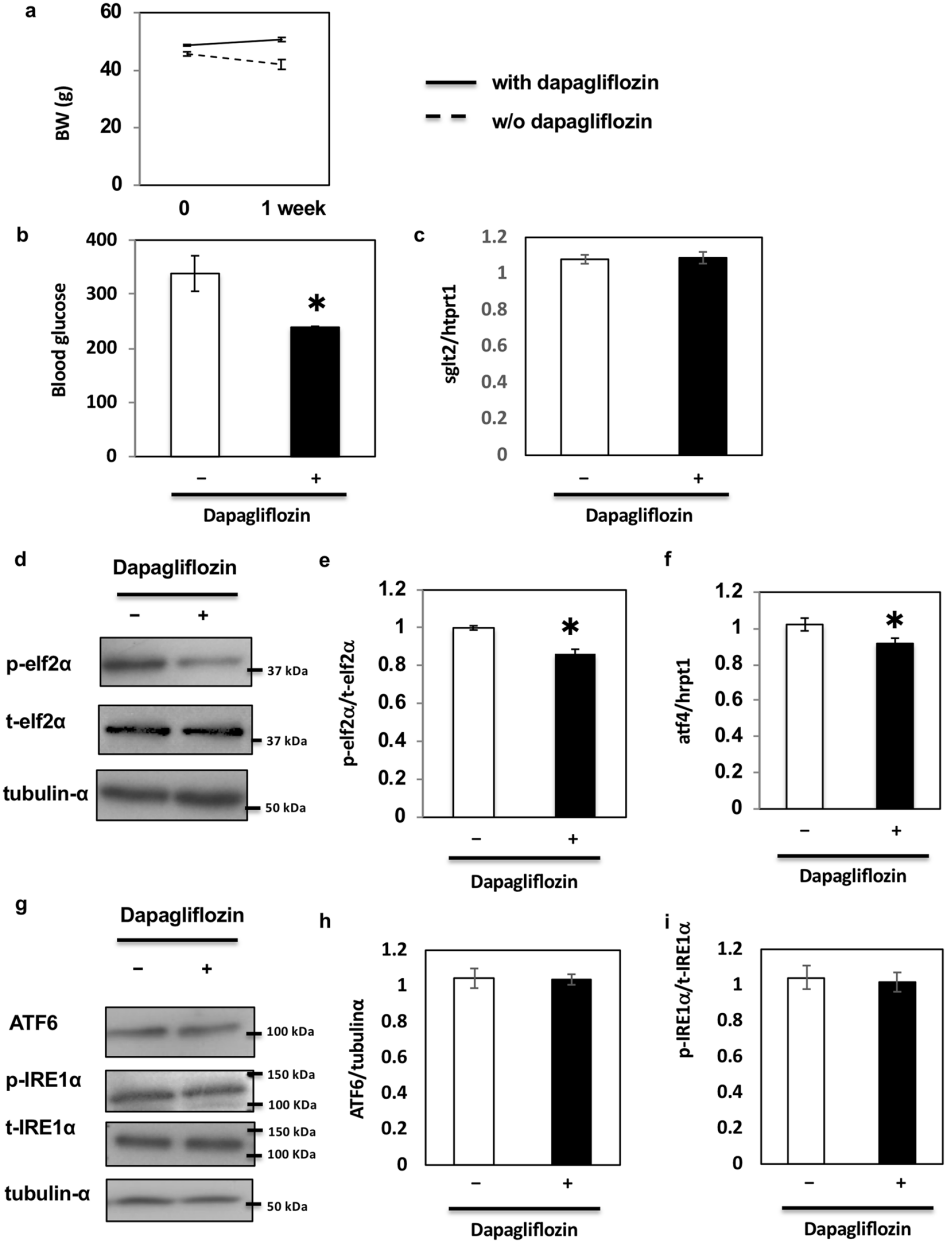
Effect of dapagliflozin on ER stress-induced cell death *in vivo*. (**a**) Body weights of db/db mice administered oral dapagliflozin every day for 1 week. (**b**) Blood glucose levels of db/db mice administered dapagliflozin. (**c**) SGLT2 mRNA levels in the kidney of db/db mice were determined by qRT-PCR (n = 5 independent experiments). (**d**,**g**) The kidneys of db/db mice were utilised for protein expression analysis. These immunoblots are representative of n = 5 experiments performed independently. (**e**) Signal quantification of the expression levels of S^51^ phosphorylation of elf2α normalised with total elf2α. (**f**) Expression levels of ATF4 mRNAs were determined by qRT-PCR (n = 5 independent experiments). (**h**,**i**) Signal quantification of the expression levels of ATF6 normalised with tubulin-α (**h**) and the expression levels of S^724^ phosphorylation of IRE1α normalised with total IRE1α (**i**).

**Figure 7:**
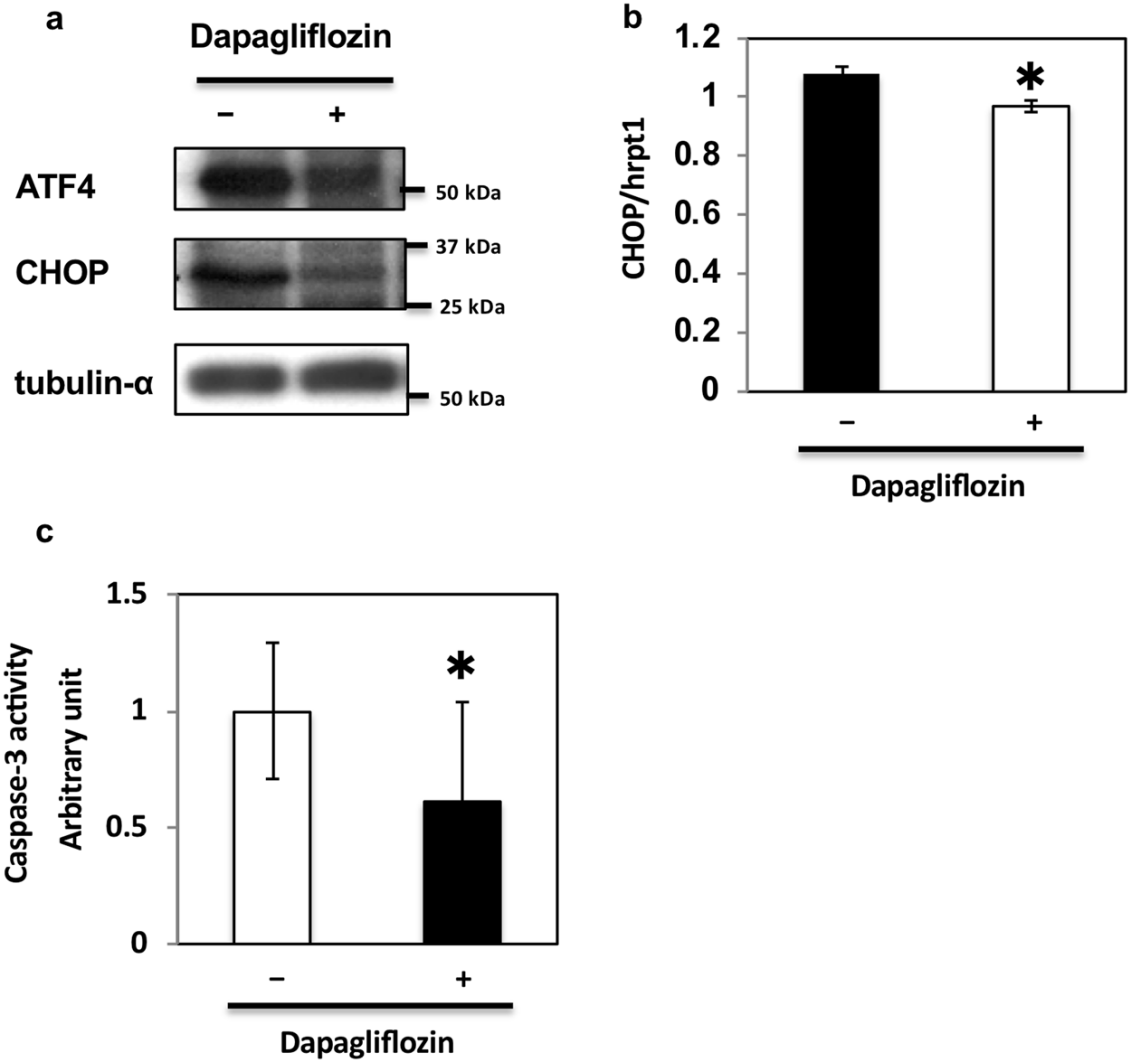
Effect of dapagliflozin on ER stress-mediated cell death *in vivo*. (**a**) The kidneys of db/db mice administered dapagliflozin were immunoblotted with indicated antibodies. (**b**) CHOP mRNA levels in the kidney of db/db mice administered dapagliflozin were determined by qRT-PCR (n = 5 independent experiments). (**c**) Caspase-3 activity levels in the kidneys of db/db mice administered dapagliflozin were determined (n = 5 independent experiments).

Our data suggested that dapagliflozin ameliorated glucose-mediated ER stress and apoptosis in HK2 cells, the model of renal proximal tubular epithelial cells *in vitro*. To further examine the effects of dapagliflozin, we repeated these experiments using kidneys of db/db mice, a mouse model of type 2 diabetes^26,27^. Previous reports have shown that ER stress is upregulated in the kidney of db/db mice^28,29^. To explore the effects of dapagliflozin on ER stress in the kidney of db/db mice, the drug was administered orally to the animals every day for 1 week. Similarly to previous studies^30,31^, we did not observe any significant decrease in body weight after only 1 week of treatment (Fig. 6a). However, blood glucose levels significantly decreased with no significant change in the expression level of SGLT2 (Fig. 6b,c). Notably, in these conditions, dapagliflozin decreased elf2α phosphorylation (Fig. 6d,e) and ATF4 expression independently of body weight change (Fig. 6f). In contrast, dapagliflozin had no effect on total and cleaved forms of ATF6 (Fig. 6g,h) or phosphorylation of IRE1α (Fig. 6g,i). ATF4 and CHOP expression and caspase-3 activity were also inhibited by dapagliflozin (Fig. 7a–c). These data indicate that dapagliflozin ameliorates ER stress in the kidney of db/db mice *in vivo* and has beneficial effects in preventing DN.

## Discussion

SGLT2 inhibitors are novel type 2 diabetes drugs that reduce blood glucose by inhibiting SGLT2 in the proximal tubules^24,25,32^. Dapagliflozin is an SGLT2 inhibitor that is rapidly absorbed after oral administration. Its maximum plasma concentration (Cmax) is approximately 0.5 µM. The working concentration of dapagliflozin has been reported to be 0.1–20 µM in HK-2 cells^33–37^. In this study, we treated HK-2 cells with 2 μM dapagliflozin based on the findings reported in previous studies.

Changes in glucose influx into the cells might contribute to the metabolic conditions that enhance ER stress in DN. Therefore, we hypothesised that inhibiting SGLT2 might reduce ER stress and protect against proximal tubular cell death. It has been reported that increasing glucose influx into cells might induce but also decrease ER stress^38–40^. In this study, we demonstrated that dapagliflozin regulates ER stress by reducing glucose concentration as well as glucose influx into HK-2 cells and that it suppresses apoptosis via regulation of the elf2α-ATF4-CHOP pathway. The fact that glucose depletion does not induce ER stress but instead ameliorates it in renal cells may appear counter-intuitive. However, several reports have indicated that renal tubular cells are easily subjected to oxidative stress and ER stress-mediated cell death. As high glucose potentially upregulates oxidative stress, this might be one plausible mechanism by which glucose depletion ameliorates ER stress in renal tubular cells^41–43^. Knockdown of elf2α counteracted the effect of dapagliflozin. It was shown that the anaesthetic drug propofol inhibits PERK-eIF2α-ATF4-CHOP activation under ER stress, caspase-3 activation, and apoptosis, whereas it activates the IRE1-XBP1 and ATF6 pathways^44^. These data indicate that the regulation of ER stress is largely dependent on the stimulus itself and pathways regulating ER stress and apoptosis vary with the nature of cells and/or pathology. Our study suggests that the elf2α-ATF4-CHOP pathway regulates ER stress in HK2 proximal tubular cells and in the kidney of db/db mice.

ER stress has been associated with many diseases including neurodegenerative disorders, type 2 diabetes, atherosclerosis, or cancer^45^. CHOP is a key protein playing a role in ER stress-induced apoptosis and one of the transcription factors regulated by both PERK-elf2α-ATF4 and ATF6^46^. IRE1α also induces apoptosis through caspases and ASK1-JNK. A positive correlation was observed between the enhanced CHOP expression and cell apoptosis in tubule epithelial cells^47^. Activation of PERK may be a key event in the regulation of CHOP expression^48^. Our findings were consistent with these data and indicated that dapagliflozin might regulate ER stress-induced apoptosis through the PERK-CHOP axis.

Ceramides are generated either by the hydrolysis of sphingomyelin catalysed by sphingomyelinase or by *de novo* synthesis in response to diverse stimuli. Under apoptotic stimuli, ceramides are generated by *de novo* synthesis and promote activation of caspases^49,50^. Notably, ceramides are induced in renal proximal tubular cells in response to hydrogen peroxide or hypoxia/reperfusion^51,52^. When ceramide generation was inhibited using SPT inhibitors and ceramide synthase inhibitors, a reduction in tubular epithelial cell death was observed^22^. Many studies on lipid metabolism in DN have been reported recently^36^. For example, AdipoRon, a therapeutic agent against DN, ameliorates renal ceramide-induced lipotoxicity in type 2 diabetes mellitus^21^. Additionally, investigating the role of ceramide is one of the major fields of study in DN, and researchers have reported excessive ceramide formation in the pathobiological condition of DN^19–22^. Therefore, we examined the effects of dapagliflozin on ER stress and apoptosis after C2 ceramide treatment and demonstrated that dapagliflozin alleviated renal cell death induced by metabolic conditions (i.e. increased glucose influx).

Recent evidence suggested that SGLT2 inhibitors not only have glucose-lowering effects but might also inhibit the progression of DN^53,54^. For example, empagliflozin, the first antidiabetic medication approved for reducing the risk of cardiovascular death in patients with type 2 diabetes, also reduces the effects of DN^54,55^. Nonetheless, further studies are necessary to investigate the specificity of dapagliflozin for reducing the effects of DN. Future studies should examine whether other SGLT2 inhibitors have potentially beneficial effects on DN as well and determine whether inhibitors of other members of the SGLT family might have similar effects on DN.

In summary, we demonstrated that dapagliflozin lowered glucose influx *in vitro* and regulated ER stress-mediated apoptosis via regulation of elf2α-ATF4-CHOP pathway. C2 ceramide treatment on HK-2 cells initiated apoptosis and lipid metabolism disorder in DN. Dapagliflozin partially reduced C2 ceramide-induced ER stress-mediated apoptosis, which might occur in DN. In addition, dapagliflozin ameliorated ER stress in the kidney of db/db mice *in vivo*. These data suggest that dapagliflozin can be used for the prevention of DN itself.

## Materials and Methods

### Antibodies and reagents

Dapagliflozin and C2 ceramide were purchased from Cayman Chemical (Ann Arbor, MI, USA) and SIGMA-ALDRICH (St. Louis, MO, USA) respectively. Rabbit polyclonal anti-elf2α, anti-phospho-elf2α Ser51, and anti-ATF4, and mouse monoclonal antibodies were from Cell Signaling Technology Inc. (Boston, MA, USA). Rabbit polyclonal anti-IRE1α and anti-phospho-IRE1α, and mouse monoclonal anti-ATF6 antibodies were from Novus Biologicals (Littleton, CO, USA). Mouse monoclonal anti-GAPDH antibody was from MBL (Woods Hole, MA, USA). All other reagents were purchased from Sigma-Aldrich.

### Culture and drug treatment *in vitro*

The human renal proximal tubular epithelial cell line HK-2 was obtained from ATCC (Manassas, VA, USA). The cells were cultured in a 100-cm^2^ dish and grown at 37 °C in an atmosphere of 5% CO_2_ in air in Keratinocyte-SFM medium supplemented with bovine pituitary extract (0.05 mg/mL) and epithelial growth factor (5 ng/mL; Gibco, Carlsbad, CA, USA). The glucose concentration of the keratinocyte-SFM medium was standard at 5.5 mmol/L. Cells were sub-cultured when they reached 70–80% confluence. Twenty-four hours after reseeding, the cells were treated either with dimethyl sulfoxide or 2 μM dapagliflozin in a serum-free culture medium for 24–48 h. As the effect of dapagliflozin was fully regulated under confluent conditions, each experiment was performed at confluence. For the D-galactose assay, HK-2 cells were incubated with the indicated concentration (0–10 mM) of D-galactose and 2 μM dapagliflozin for 48 h.

### Animals

Six male db/db mice (6 each, 10–12 weeks old, weighing around 50 g, male) were housed in a facility with a 12-h light/dark cycle and fed a standard chow diet (Research Diets, New Brunswick, NJ) containing 67% (Kcal) carbohydrates, 19% protein, and 4% fat *ad libitum*. All studies were approved by and performed in compliance with the guidelines of the institutional animal care and use committee of Gunma University. db/db mice were purchased from Charles River and acclimatised for 2 weeks. Dapagliflozin (0.5 mg/kg/day) dissolved with 0.5% methylcellulose was orally administered every day for 1 week. The mice were sacrificed by cerebral dislocation, and the kidneys were collected after fasting for 16 h followed by 4-h refeeding.

### Western blot analysis

Proteins were extracted using a lysis buffer containing a protease inhibitor as described previously^56^ and quantified using a BCA protein assay kit (Pierce, Rockford, IL, USA). Equal amounts of protein were separated using 10% sodium dodecyl sulphate–polyacrylamide gel electrophoresis and electrophoretically transferred onto nitrocellulose membranes, which were blocked with 5% fatty acid-free powdered milk for 1 h. The membranes were incubated with the following primary antibodies overnight at 4 °C: anti-p-elf2α (1:500), anti-elf2α (1:1,000), anti-IRE1 (1:1,000), anti-p-IRE1 (1:1,000), anti-ATF6 (1:200), anti-ATF4 (1:500), anti-CHOP (1:500), anti-GLUT2 (1:1,000), anti-SGLT2 (1:400), and anti-tubulin-α (1:1,000); thereafter, they were incubated with an HRP-conjugated secondary antibody for 30 min. The membranes were extensively washed in tris-buffered saline with Tween 20, and antigen-antibody complexes were visualised by chemiluminescence using an ECL kit (Pierce).

### RNA isolation and real-time qPCR

Total RNA was isolated from cells and tissues using the RNeasy Plus Mini Kit (Qiagen, Valencia, CA, USA) according to the manufacturer’s protocol. Total RNA (1,000 µg) was reverse-transcribed using the SuperScript VILO cDNA Synthesis Kit (Invitrogen, Carlsbad, CA, USA). Real-time quantitative PCR was performed on an Applied Biosystems 7500HT system (Applied Biosystems, Branchburg, NJ, USA) with EagleTaq Master Mix (Roche Lifescience). Gene expression levels of CHOP, ATF4, and SGLT2 were internally normalised against those of β actin (human) and Hrpt1 (mouse) and analysed by a standard curve method. Each experiment was performed in triplicate and independently repeated three times.

### Measurement of glucose concentration in HK2 cells

Glucose concentration was measured using the PicoProbe Glucose Fluorometric Assay Kit (BioVision, Milpitas, CA, USA). In brief, cells were lysed and proteins were incubated with the reagents provided. Samples were transferred to black-bottom 96-well microplates, and glucose concentrations were read at excitation and emission wavelengths of 535 and 587 nm, respectively, in a fluorescence microplate reader (EnSpire, Perkin Elmer). Control reactions were carried out with no protein and/or no substrate.

### Measurement of glucose influx in HK2 cells

Glucose influx was measured using a 2-Deoxyglucose Uptake Measurement Kit (COSMO BIO Co. Ltd., Tokyo Japan) according to the manufacturer’s instructions. In brief, cells were lysed and proteins were incubated with the reagents provided. Samples were transferred to 96-well microplates, and glucose influx was read at 450 nm in a kinetic microplate reader (Molecular Devices Japan). Control reactions were carried out with no protein and/or no substrate.

### Measurement of caspase-3 activity in HK2 cells

Caspase-3 activity was measured using a caspase-3 fluorescence assay kit (MBL) according to the manufacturer’s instructions. Briefly, cells and tissues were lysed and proteins were incubated with the caspase-3 substrate DEVD-AMC (2 μM) for 1 h at 37 °C. Samples were transferred to black-bottom 96-well microplates, and the relative caspase-3 activities were read at excitation and emission wavelengths of 380 and 460 nm, respectively, in a fluorescence microplate reader.

### Knockdown of elf2α in HK2 cells

After seeding of 1.0–2.5 × 10^5^ cells/12-well plates for 24 h, 100 nM siRNA for human siElF2A-lipid complexes with DharmafectDuo (Dharmacon/Thermo Scientific, Chicago, IL) was introduced into each well. After 24 h, the medium was replaced with dimethyl sulfoxide or 2 µM dapagliflozin and incubated for an additional 24 h. Knockdown efficiencies were confirmed by western blot analysis or real-time qPCR.

### Apoptosis and necrosis assay in HK2 cells

An apoptosis and necrosis assay was performed using Cell Death Detection ELISAplus (Roche, Indianapolis, IN) according to the manufacturer’s instructions. In brief, after seeding of 1.0 × 10^5^ cells/96-well plates for 24 h, HK-2 cells were incubated with 7.5 ng/mL C2 ceramide and 2 µM dapagliflozin. After 48 h, the cells were harvested to perform apoptosis and necrosis assay.

### Statistics

The results are expressed as mean ± standard error of the mean. Differences between cells and/or treatments were tested for statistical significance (P < 0.05) using Student’s unpaired *t* test and ANOVA multiple comparisons using Tukey’s honestly significant difference test.

## Supplementary information

supplement figure

## Data Availability

The authors confirm that the data supporting the findings of this study are available within the article and its supplementary materials.

## References

[1] Tuomilehto J (2001). Prevention of type 2 diabetes mellitus by changes in lifestyle among subjects with impaired glucose tolerance. N. Engl. J. Med..

[2] Orozco LJ (2008). Exercise or exercise and diet for preventing type 2 diabetes mellitus. Cochrane Database Syst. Rev..

[3] Baik J, Nguyen D, Nguyen V, Hu Z, Abbott GW (2017). Kcne2 deletion impairs insulin secretion and causes type 2 diabetes mellitus. FASEB J..

[4] Costantino L, Rastelli G, Vianello P, Cignarella G, Barlocco D (1999). Diabetes complications and their potential prevention: aldose reductase inhibition and other approaches. Med. Res. Rev..

[5] Fan Y, Lee K, Wang N, He JC (2017). The role of endoplasmic reticulum stress in diabetic nephropathy. Curr. Diab. Rep..

[6] Cunard R (2015). Endoplasmic reticulum stress in the diabetic kidney, the good, the bad and the ugly. J. Clin. Med..

[7] Cao SS, Kaufman RJ (2012). Unfolded protein response. Curr. Biol..

[8] Ellgaard L, Molinari M, Helenius A (1999). Setting the standards: quality control in the secretory pathway. Science.

[9] Harding HP, Zhang Y, Bertolotti A, Zeng H, Ron D (2000). Perk is essential for translational regulation and cell survival during the unfolded protein response. Mol. Cell..

[10] Huber AL (2013). p58(IPK)-mediated attenuation of the proapoptotic PERK-CHOP pathway allows malignant progression upon low glucose. Mol. Cell..

[11] Chen X, Shen J, Prywes R (2010). The luminal domain of ATF6 senses endoplasmic reticulum (ER) stress and causes translocation of ATF6 from the ER to the Golgi. J. Biol. Chem..

[12] Urano F (2000). Coupling of stress in the ER to activation of JNK protein kinases by transmembrane protein kinase IRE1. Science.

[13] Liu G (2008). Apoptosis induced by endoplasmic reticulum stress involved in diabetic kidney disease. Biochem. Biophys. Res. Commun..

[14] Basha B, Samuel SM, Triggle CR, Ding H (2012). Endothelial dysfunction in diabetes mellitus: possible involvement of endoplasmic reticulum stress?. Exp. Diabetes Res..

[15] Zhong Y (2012). Activation of endoplasmic reticulum stress by hyperglycemia is essential for Müller cell-derived inflammatory cytokine production in diabetes. Diabetes.

[16] Ponnusamy S (2010). Sphingolipids and cancer: ceramide and sphingosine-1-phosphate in the regulation of cell death and drug resistance. Future Oncol..

[17] Movsesyan VA, Yakovlev AG, Dabaghyan EA, Stoica BA, Faden AI (2002). Ceramide induces neuronal apoptosis through the caspase-9/caspase-3 pathway. Biochem. Biophys. Res. Commun..

[18] Haus JM (2009). Plasma ceramides are elevated in obese subjects with type 2 diabetes and correlate with the severity of insulin resistance. Diabetes.

[19] Yaribeygi, H, Bo, S, Ruscica, M & Sahebkar, A. Ceramides and diabetes mellitus: an update on the potential molecular relationships. *Diabet Med*. *Feb* **25** (2019). 10.1111/dme.13943 30803019

[20] Galadari, S *et al*. Role of ceramide in diabetes mellitus: evidence and mechanisms. *Lipids in Health and Disease* **12****(****98****)** (2013). 10.1186/1476-511X-12-98 PMC3716967 23835113

[21] Srivastava, S. P. *et al*. Lipid mediators in diabetic nephronpathy. *Fibrogenesis Tissue Repair*. **7**–**12** (2014). 10.1186/1755-1536-7-12 PMC4159383 25206927

[22] Choi SR (2018). Adiponectin receptor agonist AdipoRon decreased ceramide, and lipotoxicity, and ameliorated diabetic nephronpathy. Metabolism..

[23] Bonventre JV (2012). Can we target tubular damage to prevent renal function decline in diabetes?. Semin. Nephrol..

[24] Mosley JF, Smith L, Everton E, Fellner C (2015). Sodium-glucose linked transporter 2 (SGLT2) inhibitors in the management of type-2. diabetes: a drug class overview. P T..

[25] Gnudi L, Coward RJ, Long DA (2016). Diabetic nephropathy: perspective on novel molecular mechanisms. Trends Endocrinol. Metab..

[26] Zhang, J., Fan, Y., Zeng, C., He, L. & Wang, N. Tauroursodeoxycholic acid attenuates renal tubular injury in a mouse model of type 2 diabetes. *Nutrients* **8****(****10****)** (2016). 10.3390/nu8100589 PMC5083977 27669287

[27] Cao AL (2016). Ursodeoxycholic acid and 4-phenylbutyrate prevent endoplasmic reticulum stress-induced podocyte apoptosis in diabetic nephropathy. Lab. Invest..

[28] Fan Y (2017). Rtn1a-Mediated Endoplasmic Reticulum Stress in Podocyte Injury and Diabetic Nephropathy. Sci Rep..

[29] Chen J (2014). stress triggers MCP-1 expression through SET7/9-induced histone methylation in the kidneys of db/db mice. Am J Physiol Renal Physiol..

[30] Jia Y (2018). Dapagliflozin Aggravates Renal Injury via Promoting Gluconeogenesis in db/db Mice. Cellular Physiology and Biochemistry.

[31] Terami Naoto, Ogawa Daisuke, Tachibana Hiromi, Hatanaka Takashi, Wada Jun, Nakatsuka Atsuko, Eguchi Jun, Horiguchi Chikage Sato, Nishii Naoko, Yamada Hiroshi, Takei Kohji, Makino Hirofumi (2014). Long-Term Treatment with the Sodium Glucose Cotransporter 2 Inhibitor, Dapagliflozin, Ameliorates Glucose Homeostasis and Diabetic Nephropathy in db/db Mice. PLoS ONE.

[32] Bakris GL, Fonseca VA, Sharma K, Wright EM (2009). Renal sodium-glucose transport: role in diabetes mellitus and potential clinical implications. Kidney Int..

[33] Komoroski, B. *et al*. Dapagliflozin, a Novel SGLT2 Inhibitor, Induces Dose‐Dependent Glucosuria in Healthy Subjects. Clinical Pharmacology & Therapeutics (2009) 10.1038/clpt.2008.251 19129748

[34] Kasichayanula S (2014). Clinical Pharmacokinetics and Pharmacodynamics of Dapagliflozin, a Selective Inhibitor of Sodium-Glucose Co-transporter Type 2. Clinical Pharmacokinetics.

[35] Lim Jae Cheong, Lim Seul Ki, Park Min Jung, Kim Gye Yeop, Han Ho Jae, Park Soo Hyun (2011). Cannabinoid receptor 1 mediates high glucose-induced apoptosis via endoplasmic reticulum stress in primary cultured rat mesangial cells. American Journal of Physiology-Renal Physiology.

[36] Chang Yoon-Kyung, Choi Hyunsu, Jeong Jin Young, Na Ki-Ryang, Lee Kang Wook, Lim Beom Jin, Choi Dae Eun (2016). Correction: Dapagliflozin, SGLT2 Inhibitor, Attenuates Renal Ischemia-Reperfusion Injury. PLOS ONE.

[37] Lu YT (2019). A Fluorescent Glucose Transport Assay for Screening SGLT2 Inhibitors in Endogenous SGLT2-Expressing HK-2 Cells. Nat Prod Bioprospect..

[38] Kothinti RK, Blodgett AB, North PE, Roman RJ, Tabatabai NM (2012). A novel SGLT is expressed in the human kidney. Eur J Pharmacol..

[39] Karunakaran U (2019). Myricetin Protects Against High Glucose-Induced β-Cell Apoptosis by Attenuating Endoplasmic Reticulum Stress via Inactivation of Cyclin-Dependent Kinase 5. Diabetes Metab J..

[40] Ma L (2019). Low glucose and metformin-induced apoptosis of human ovarian cancer cells is connected to ASK1 via mitochondrial and endoplasmic reticulum stress-associated pathways. J Exp Clin Cancer Res.

[41] Chang JW (2016). Up-Regulation of SIRT1 Reduces Endoplasmic Reticulum Stress and Renal Fibrosis. Nephron..

[42] Jiang X (2019). Overexpression of augmenter of liver regeneration (ALR) mitigates the effect of H(2)O(2)-induced endoplasmic reticulum stress in renal tubule epithelial cells. Apoptosis..

[43] Lin M (2014). Baicalin ameliorates H2O2 induced cytotoxicity in HK-2 cells through the inhibition of ER stress and the activation of Nrf2 signaling. Int J Mol Sci..

[44] Zhou X (2016). Propofol decreases endoplasmic reticulum stress-mediated apoptosis in retinal pigment epithelial cells. PLoS One.

[45] Ozcan L, Tabas I (2012). Role of endoplasmic reticulum stress in metabolic disease and other disorders. Annu. Rev. Med..

[46] Zhang K, Kaufman RJ (2008). From endoplasmic-reticulum stress to the inflammatory response. Nature.

[47] Wu X, He Y, Jing Y, Li K, Zhang J (2010). Albumin overload induces apoptosis in renal tubular epithelial cells through a CHOP-dependent pathway. OMICS.

[48] Wang M, Kaufman RJ (2014). The impact of the endoplasmic reticulum protein-folding environment on cancer development. Nat. Rev. Cancer.

[49] Ogretmen B, Hannun YA (2004). Biologically active sphingolipids in cancer pathogenesis and treatment. Nat. Rev. Cancer.

[50] Ueda N, Kaushal GP, Hong X, Shah SV (1998). Role of enhanced ceramide generation in DNA damage and cell death in chemical hypoxic injury to LLC-PK1 cells. Kidney Int..

[51] Basnakian AG (2005). Ceramide synthase is essential for endonuclease-mediated death of renal tubular epithelial cells induced by hypoxia-reoxygenation. Am. J. Physiol. Renal Physiol..

[52] Komoroski B (2009). Dapagliflozin, a novel, selective SGLT2 inhibitor, improved glycemic control over 2 weeks in patients with type 2 diabetes mellitus. Clin. Pharmacol. Ther..

[53] Wanner, C. *et al*. EMPA-REG OUTCOME Investigators. Empagliflozin and Progression of Kidney Disease in Type 2 Diabetes. *N Engl J Med*. Jul **28**;**375**(**4**), 323–34 (2016). 10.1056/NEJMoa1515920 27299675

[54] Neal, B. *et al*. CANVAS Program Collaborative Group. Canagliflozin and Cardiovascular and Renal Events in Type 2 Diabetes. *N Engl J Med*. Aug **17**;**377**(**7**), 644–657(2017). 10.1056/NEJMoa1611925 28605608

[55] Zinman B (2015). Empagliflozin, cardiovascular outcomes, and mortality in type 2 diabetes. N. Engl. J. Med..

[56] Yamada E (2016). Fyn phosphorylates AMPK to inhibit AMPK activity and AMP-dependent activation of autophagy. Oncotarget.

