# Supplementary material for: Dapagliflozin rescues endoplasmic reticulum stress-mediated cell death
Source: Sci Rep. 2019 Jul 8;9:9887. doi: 10.1038/s41598-019-46402-6 (PMC6614429; doi:10.1038/s41598-019-46402-6)
Supplement: Supplementary file 1 — supplement figure [file 41598_2019_46402_MOESM1_ESM.pdf]

Dapagliflozin rescues endoplasmic reticulum stress-mediated cell death

Ryo Shibusawa[1], Eijiro Yamada[1*], Shuichi Okada[1], Yasuyo Nakajima[1], Claire C Bastie[2], Akito Maeshima[3], Kyoichi Kaira[4], and Masanobu Yamada[1]

[1]Department of Medicine and Molecular Science, [4]Department of Oncology Clinical Development, Gunma University Graduate School of Medicine, Maebashi 371-8511, Japan

[2]Division of Biomedical Sciences, Warwick Medical School, Coventry, West Midlands, United Kingdom

[3]Division of Nephrology, Department of Internal Medicine, Jichi Medical University, Shimotsuke, Tochigii, Japan

Running title: Dapagliflozin decreases ER stress and apoptosis

Keywords: SGLT2, ER stress, apoptosis

*Correspondence to:

Eijiro Yamada

Department of Medicine and Molecular Science, Gunma University Graduate School of Medicine, Maebashi 371-8511, Japan

Email: eijiro.yamada@gunma-u.ac.jp

Phone: 1-81-27-220-8501

FAX: 1-81-27-220-8136

Supplementary Fig. 1: Different concentrations of HK2 cells for p-eIF2a/t-eIF2a analysis

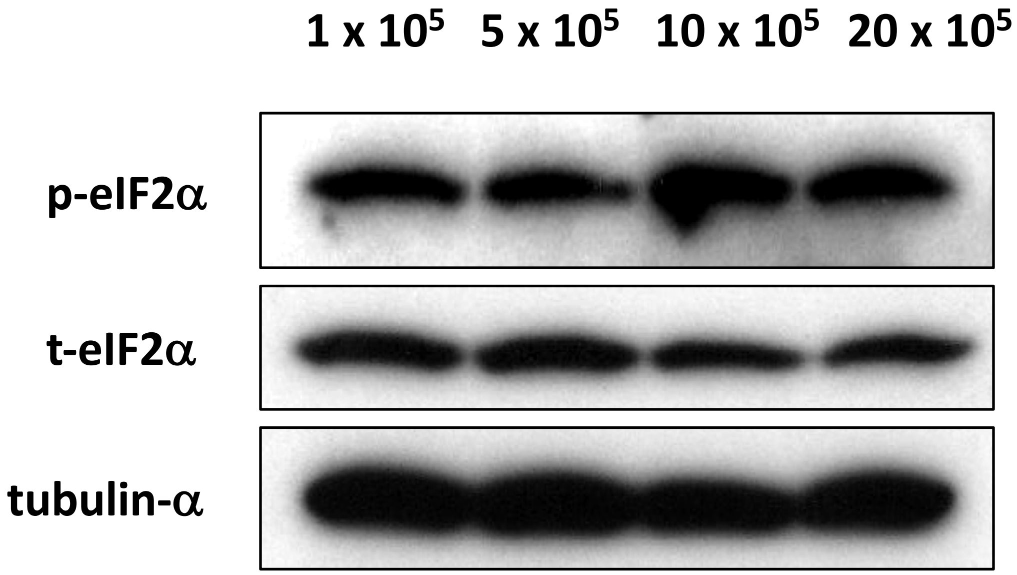

Figure Legend: Different cell numbers of HK2 cells were plated on to a 24-well plate. Immunoblotting was performed with the indicated antibodies.

Supplementary Fig. 2

a) Western blots cropped for Figure 2a

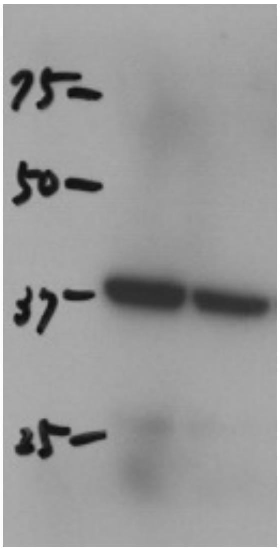

p-elf2α

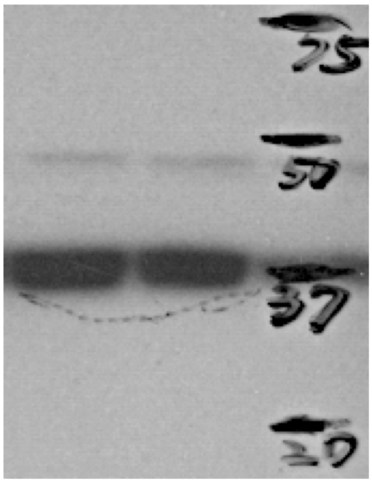

t-elf2α

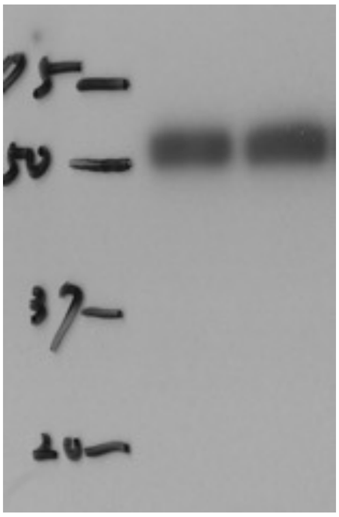

tubulin-α

b) Western blots cropped for Figure 2d

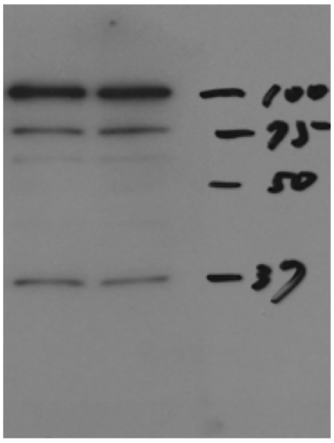

ATF6

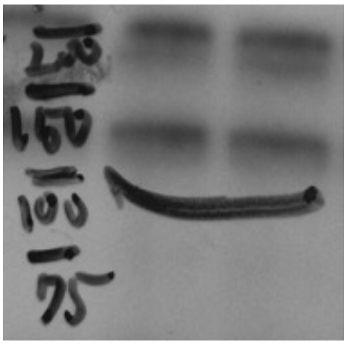

p-IRE1α

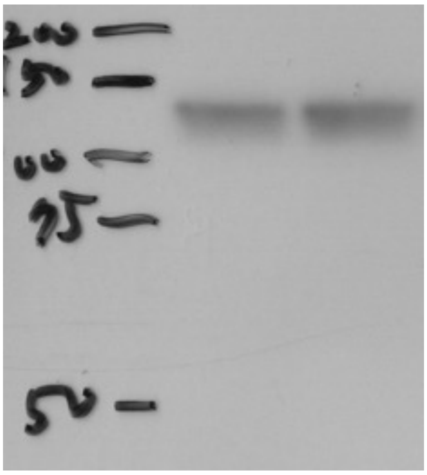

t-IRE1α

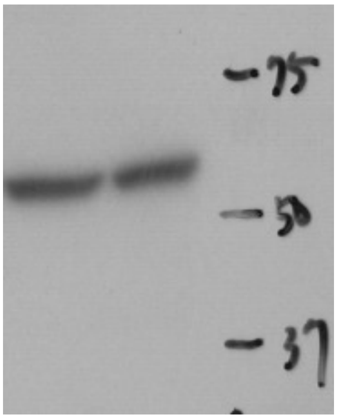

tubulin-α

Supplementary Fig. 3

a) Western blots cropped for Figure 3a

p-eIf2α

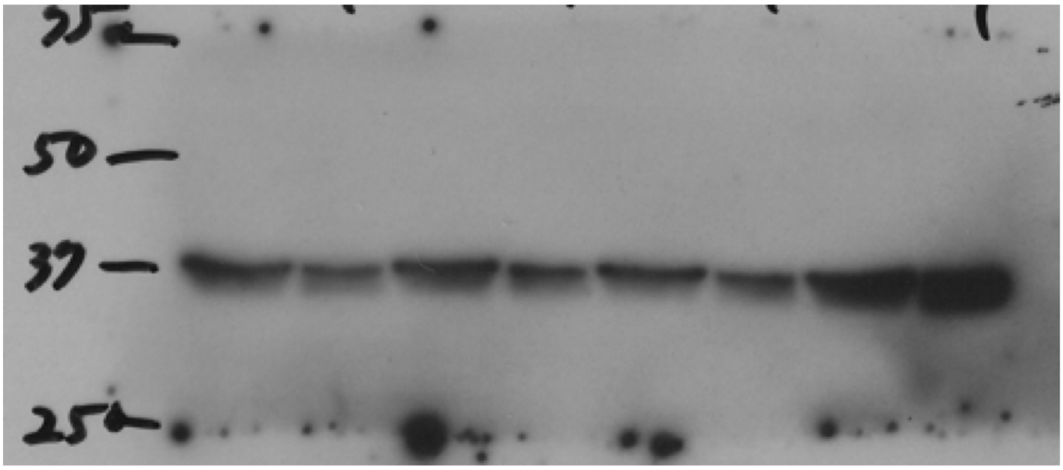

t-eIf2α

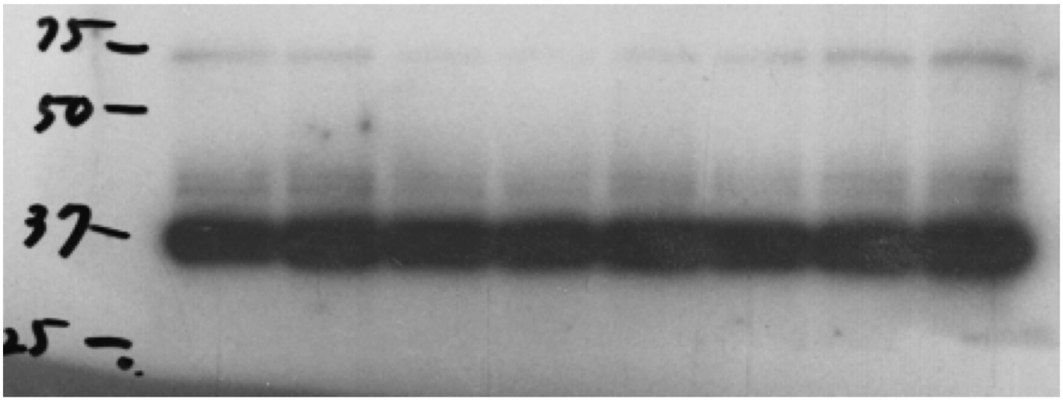

tubulin-α

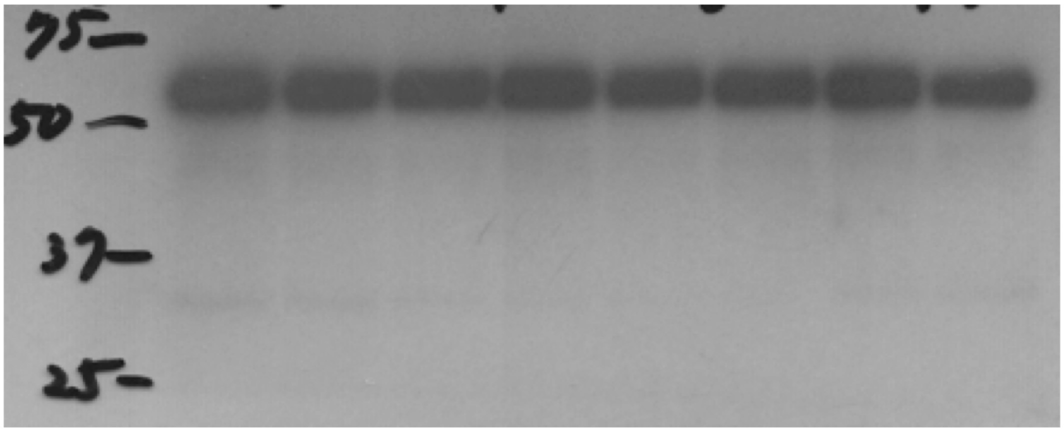

Supplementary Fig. 4

a) Western blots cropped for Figure 3b

p-elf2α

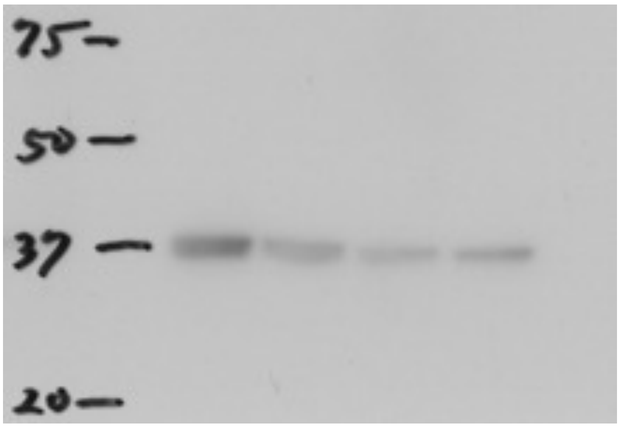

t-elf2α

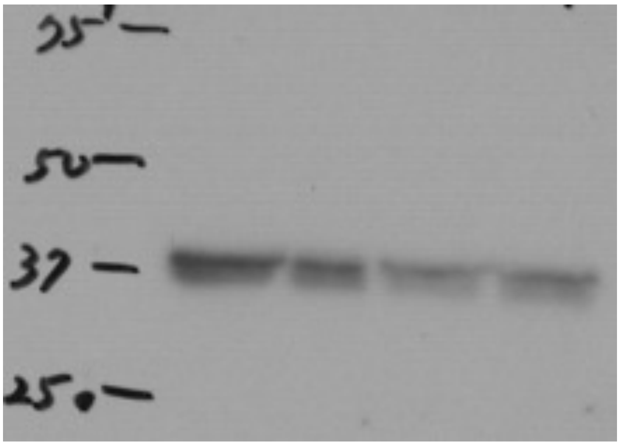

tubulin-α

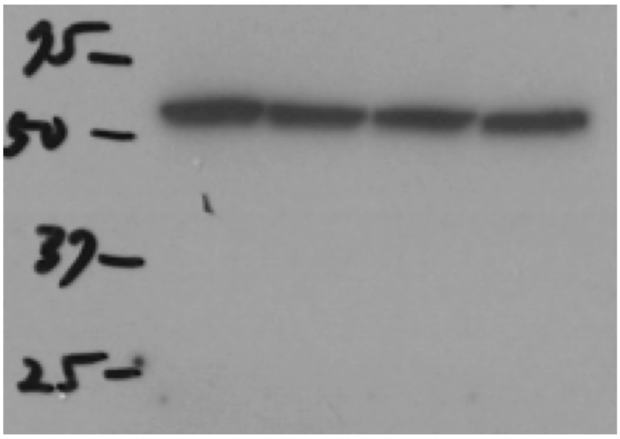

Supplementary Fig. 5

a) Western blots cropped for Figure 4a

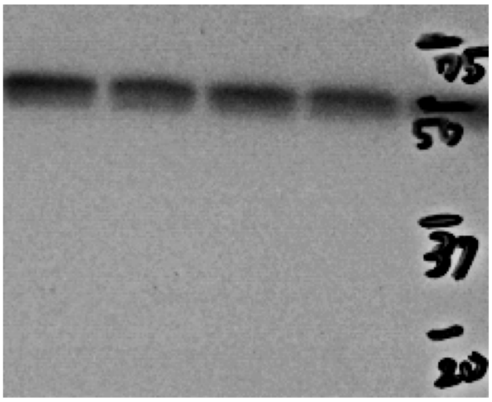

GLUT2

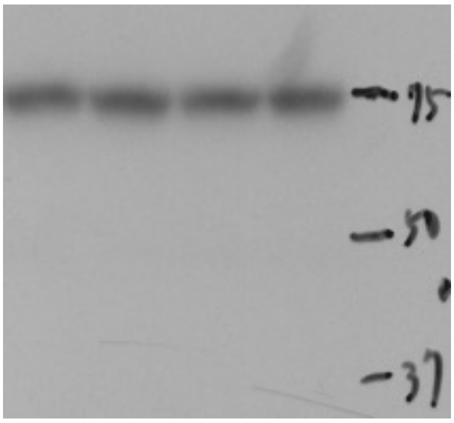

SGLT2

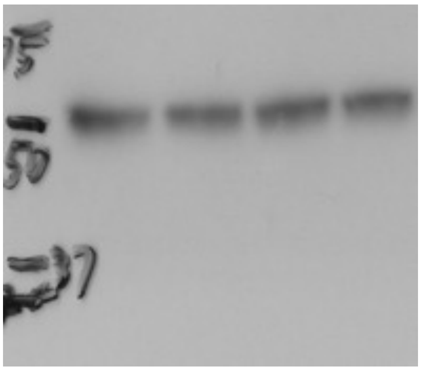

tubulin-α

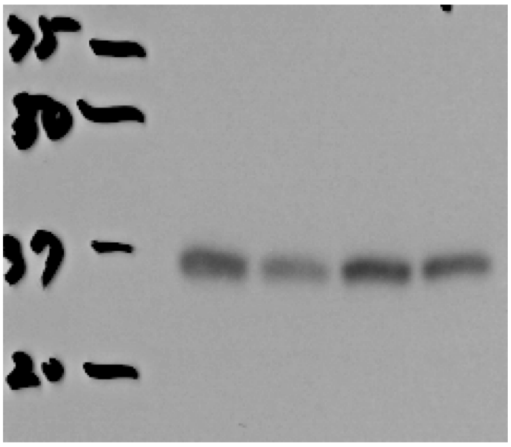

p-elf2α

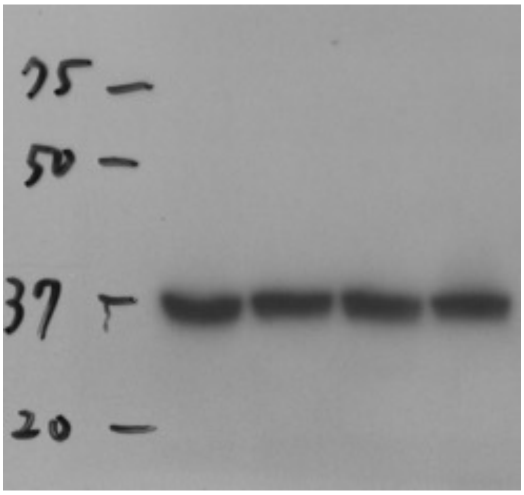

t-elf2α

Supplementary Fig. 6

a) Western blots cropped for Figure 6d

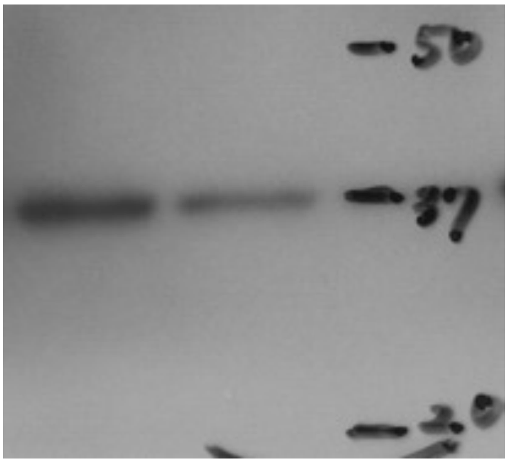

p-elf2α

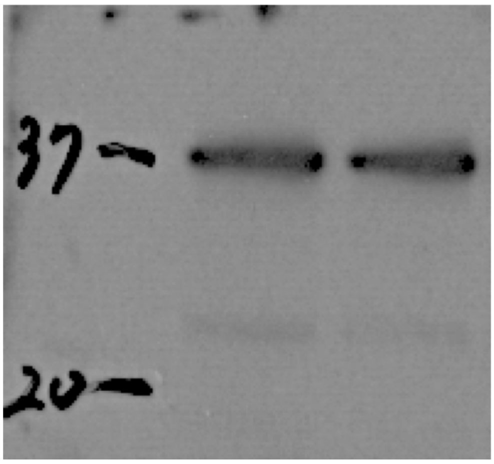

t-elf2α

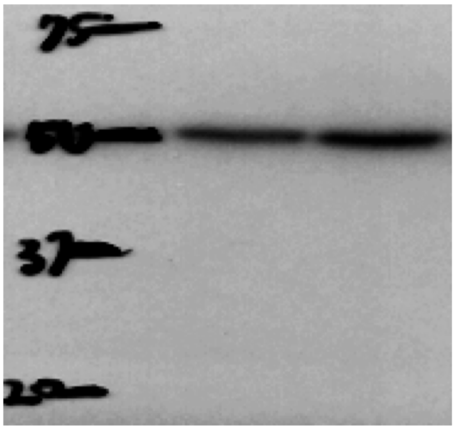

tubulin-α

b) Western blots cropped for Figure 6g

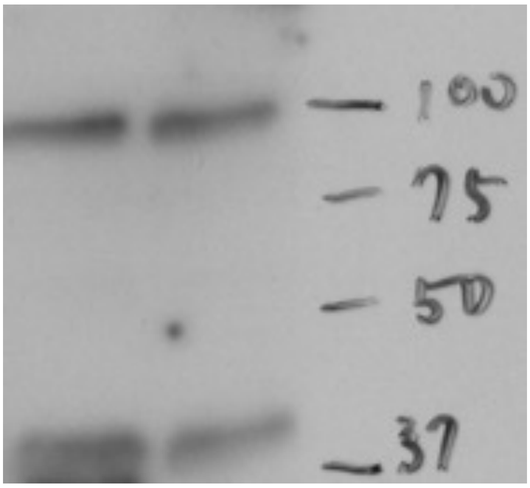

ATF6

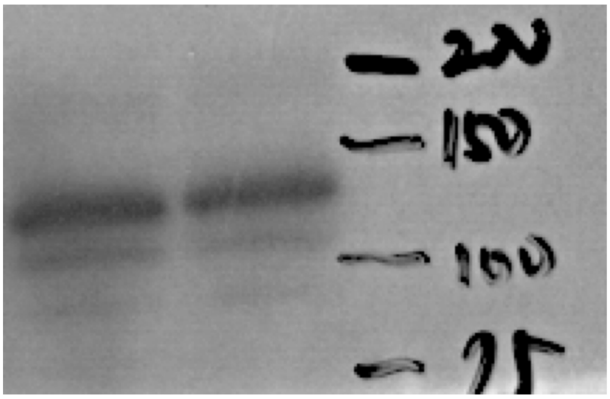

p-IRE1α

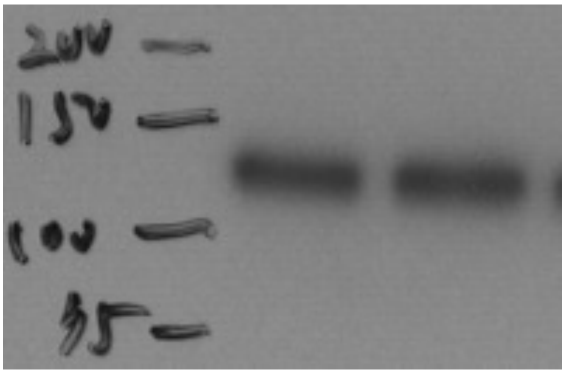

t-IRE1α

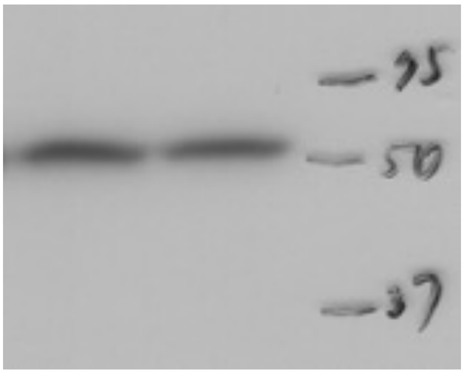

tubulin-α

Supplementary Fig. 7

a) Western blots cropped for Figure 7a

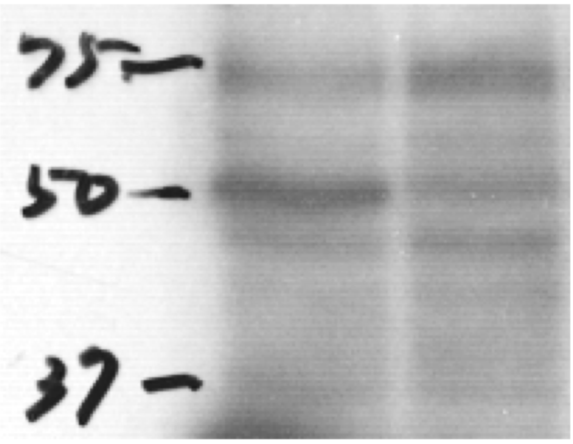

ATF4

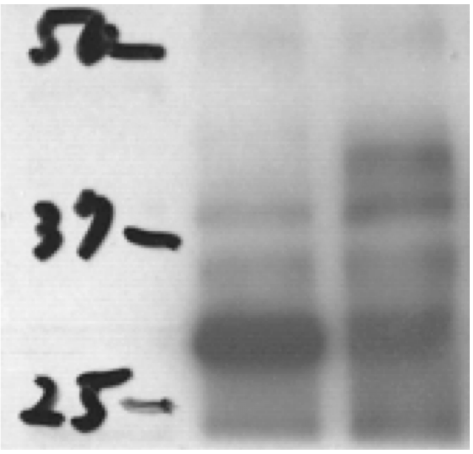

CHOP

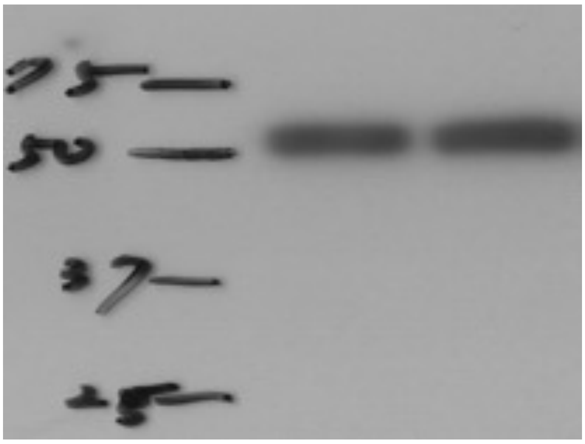

tubulin-α
